# Remote Management for Multipoint Sensing Systems Using Hetero-Core Spliced Optical Fiber Sensors

**DOI:** 10.3390/s140100468

**Published:** 2013-12-27

**Authors:** Lee See Goh, Yuji Anoda, Watanabe Kazuhiro, Norihiko Shinomiya

**Affiliations:** Graduate School of Engineering, Soka University, 1-236, Tangi-machi, Hachioji-shi, Tokyo 192-8577, Japan; E-Mails: gohleesee@soka.gr.jp (L.S.G.); happy-go-lucky_36@soka.gr.jp (Y.A.); kazuhiro@soka.ac.jp (W.K.)

**Keywords:** optical fiber sensor, hetero-core optical fiber sensors, surface plasmon resonance sensors, bending sensors, multipoint sensing, remote management, internet standard protocol

## Abstract

This paper describes the design and experimental verification of a multipoint sensing system with hetero-core spliced optical fiber sensors and its remote management using an internet-standard protocol. The study proposes two different types of design and conducts experiments to verify those systems' feasibility. In order to manage the sensing systems remotely, the management method uses a standard operation and maintenance protocol for internet: the Simple Network Management Protocol is proposed. The purpose of this study is to construct a multipoint sensing system remote management tool by which the system can also determine the status and the identity of fiber optic sensors. The constructed sensing systems are verified and the results have demonstrated that the first proposed system can distinguish the responses from different hetero-core spliced optical fiber sensors remotely. The second proposed system shows that data communications are performed successfully while identifying the status of hetero-core spliced optical fiber sensors remotely.

## 1. Introduction

In recent years, studies focusing on sensor networks have attracted the interest and attention of many researchers. These sensor networks act as information infrastructures, helping to provide ubiquitous services by using the information from daily life [1–3].

Although many such wireless sensor networks (WSNs) seem to be successfully deployed and have evolved in many aspects, they continue to be networks with constrained resources in terms of limited power, memory, and computational capacities [4,5]. Power efficiency is the main concern in sensor networks; however, the Quality of Service (QoS) requirements also need to be satisfied [6]. In the study by Zhu *et al.*, they mentioned that coverage is one of the measurements of WSN QoS and it is closely related to energy consumption [7]. In addition, nodes have limited communication capabilities, when a source node can only cover the area within its maximum transmission range [8].

Optical fiber, which has been developed for high-speed data transmission, has also been employed as a sensor for remote data monitoring of environmental conditions or physical properties [9–11]. In optical fiber sensing systems, fiber sensor elements use light propagating along optical fibers to take measurements. For that reason, optical fiber sensors do not need secondary power supplies, although related data communications and measurement equipment may. Additionally, using optical fiber as a transmission medium allows higher speed and larger data communications.

An optical fiber sensing system that could utilize the benefits that optical fiber offers to both data communications and sensing would likely resolve many existing issues. This paper proposes two types of optical fiber sensing systems that use hetero-core spliced (HC) optical fiber sensors. These sensors can be easily manufactured by a simple cutting and fusion splicing process; they have been evaluated positively in previous research for their sensitivity and the high measurement precision [12–15].

In the review by Rathnayaka and Potdar [16], transport protocols for WSNs are discussed. Due to the numerous requirements and constraints on WSNs, many standard network transport protocols such as User Datagram Protocol (UDP) and Transmission Control Protocol (TCP) are not appropriate.

To monitor sensor conditions in the system, an existing internet-standard protocol which works in the application layer of the Open Systems Interconnection (OSI) model is used. The objective of this study is to install multiple sensors into one transmission line, remotely manage them and differentiate the response from each sensor by using the Simple Network Management Protocol (SNMP).

In Section 2 of this paper, details of the hetero-core optical fiber sensors, results from previous studies, and remaining issues are described. In Section 3, two proposals for remote management of optical fiber sensing systems that use SNMP are presented and the evaluation test results are discussed. Lastly, the paper is summarized and future research directions are discussed.

## 2. Hetero-Core Optical Fiber Sensor System

### 2.1. HC Optical Fiber Sensors

An HC fiber optic sensor consists of a transmission line and a spliced hetero-core portion which works as a sensor. These sensors are made from different types and core diameters of optical fiber. In this study, two types of sensor modules are used: HC surface plasmon resonance (HC-SPR) sensors and HC bending sensors.

#### 2.1.1. SPR Sensors

**Figure 1.**
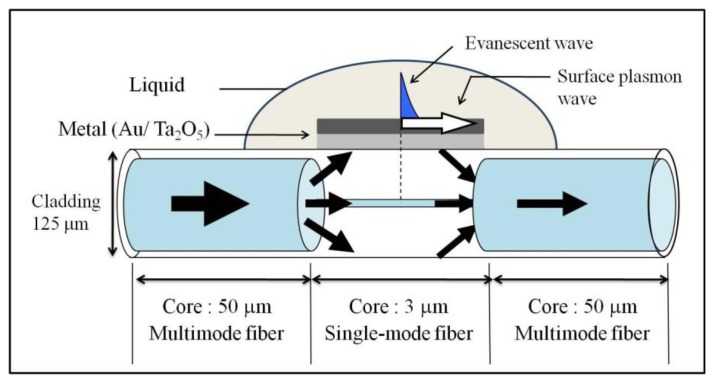
Structure of the hetero-core structured optical fiber SPR sensor.

A multimode fiber and a single-mode fiber are used to construct an HC-SPR sensor. Figure 1 shows the structure of the HC-SPR sensor used in this study. This sensor type is fabricated with a graded index multimode fiber and a step index single-mode fiber. The core diameter of the multi-mode fiber is 50 μm and the core of single-mode fiber is 3 μm. The multi-mode fiber works as transmission line, while the single-mode fiber acts as a sensor. The length of the sensor portion can range from 2 to 20 mm. The core diameter of the inserted fiber is much smaller than the transmission line. Consequently, most of the light wave will leak into the cladding layer at the interface of the transmission line and the hetero-core portion.

After the HC optical fiber has been prepared, the HC-SPR portion is then fabricated by uniformly coating the bare fiber surface with metal using a radio frequency (RF) sputtering machine (CFS-4DS-231; Shibaura Mechatronics Corp., Yokohama, Japan). The metallic coating on the cylindrical surface of the hetero-core portion allows the formation of a surface plasmon wave when an evanescent wave reflects on the metal surface. The light leakage generates an evanescent wave in the cladding when it reflects at the boundary surface between the cladding and the surrounding medium [13].

The SPR resonance wavelength can vary because it depends on the refractive index and absorbance of the coated metal surface. Thus, this mechanism can be used as a sensor for measuring refractive index by measuring changes in the resonance wavelength.

However, in this study, the light leakage that causes SPR resonance in the HC-SPR sensor is measured as light loss. Therefore, this system measures changes in signal intensity.

#### 2.1.2. Bending Sensors

Bending sensors can be served as binary switch sensors. The data transmission line and the sensor portion are made by single-mode fiber. The transmission line has a 9 μm core diameter; the sensor portion has a 5 μm core. The length of the sensor portion can range from 1 to 10 mm. The HC sensor portion diameter is smaller than the transmission portion. As in HC-SPR sensors, a small amount of light is leaked when it travels through the sensor portion. The sensor works by a light leakage mechanism, so the light loss is measured. This concept can also be used to develop a binary switch module.

**Figure 2.**
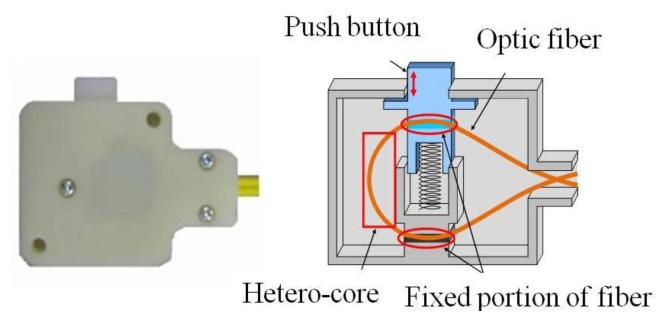
Structure of the binary switch module.

Figure 2 shows a schematic of a binary switch module developed for insertion into a communication line. The HC optical fiber is bent to a certain degree when assembling the module. The push button causes a slight change in the bending of the hetero-core portion. The changes in light leakage from this sensor depend on the changes induced in bending pattern by toggling the switch. Therefore, this switch module is appropriate for monitoring binary information.

### 2.2. Soil Gravity Water Monitoring

In previous research, an HC-SPR sensor system was constructed and verification tests were conducted [17]. That study employed HC-SPR sensors coated with 25 nm thickness of gold (Au) and 60 nm thickness of tantalum pentoxide (Ta_2_O_5_); these sensors were used specifically for soil gravitational water detection. In order to provide real-time measurement data to users, integrating communication and measurement devices in the system with cloud services has been studied. This sensor system construction has been tested, and the system successfully and simultaneously gathered sensor data and viewed a web camera without problems.

### 2.3. Optical Fiber Sensor Network Integrating Data Communications and Sensing

An optical fiber sensor network has been constructed and evaluated for its data communications and sensing performance by Abe *et al.* [3]. The bending sensor, made of single-mode optical fiber, was employed in that study. The monitoring space and optical sensory nerve network concept was described, and a trial environment was constructed and evaluated for feasibility. The system was constructed based on requirements such as low cost, simple configuration, communication quality, measurement precision, and multiplicity (*i.e.*, the number of sensors on each optic fiber). The sensing performance and multiplicity of the proposed sensor network was described. The evaluation results demonstrated that data communications and sensing could be concurrently realized.

### 2.4. Remaining Issues of Previous Research and System Requirements

In the study of soil gravitational water monitoring, described in Section 2.2, the sensing data was successfully gathered remotely during video data transmission. Another study described in Section 2.3 has successfully implemented a multipoint sensor test with binary switch sensors. However, remote management for these sensing systems has not yet been studied. Thus, this paper studied and constructed two different types of internet-based remote data acquisition sensing systems by using SNMP. The details of these two proposed sensing systems and the requirements for remote management are presented next.

## 3. Remote Management for Optical Sensing System by SNMP

In order to manage a multipoint optical fiber sensing systems remotely the following functions are required: (i) the ability to distinguish the identity of responding sensors and (ii) real-time measurement and data transmission.

Vancea and Dobrota [18] discussed a related topic, a sensor network management of WSN devices by SNMP with IEEE 802.15.4. However, their work is intended to provide a system that can be used for management of low-rate wireless personal area network equipment.

In the first proposal of this study, the system requires to identify the response from multiple sensors installed into the same fiber line, while, the second proposal is considering both the attenuation of light by HC optical fiber sensors as an element to measure the sensing performance and the value of the light intensity as a performance indicator for the communication signal among the resources to be monitored. This proposed system use the attenuation of light intensity in the data communications signal to measure the sensing performance; therefore, it makes sense to consider failures in data communications as arising from attenuation of the light. These sensing systems are constructed by installing multiple HC optical fiber sensors in the same fiber line. To manage this, a method that relies on SNMP is used. SNMP can detect changes in the network environment by means of a threshold value and the use of trap functions. To identify the operational status of multiple sensors, a unique management information base (MIB) must be defined and used by the SNMP agent and SNMP manager. SNMP agent device used in this study can act as measuring devices for sensor data and also as an SNMP agent. This device is connected to an Ethernet interface, and four-channel analog input to this device can be used to measure electric voltage which converted from the light intensity responded from the sensor.

The SNMP agent requires a script to function. Before writing the script into the SNMP agent, the output value of the sensors which are tandem connected in fiber line are measured, in a few combinations. After identifying the different output values from the sensors, the trap numbers are assigned to each value in the script. Then, the SNMP manager will refer to the trap numbers to identify the source of the response from the tandem connected sensors.

Two different system configurations and experiments for the remote management for multipoint optical fiber sensing systems are discussed next.

### 3.1. Identification of SPR Sensors

#### 3.1.1. System Configuration for Multipoint HC-SPR Sensors

**Figure 3.**
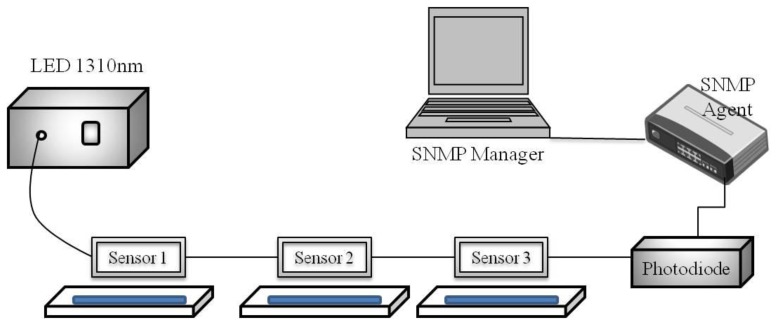
System configuration of sensing system remote management using the SNMP for HC-SPR sensors.

Takagi and Watanabe [19] studied multipoint water detection using HC-SPR sensors. Their detection scheme employed a combination of an LED (light-emitting diode) light source and a photodiode. However, the sensing system remote management is not implemented in that study. This study extends the application of the above previous work and the proposed system configuration is shown in Figure 3. Takagi and Watanabe [19] discussed that SPR spectrum changes with various refractive indices of liquids for the 25/60 coating under the condition that the resonant wavelength appeared around 1,300 nm. Therefore, this study uses 25 nm Au and 60 nm Ta_2_O_5_-coated HC-SPR sensors. Three HC-SPR sensors with different insertion lengths are used. The insertion lengths of the sensors are 2, 5 and 15 mm for sensors 1, 2 and 3, respectively. The system devices are an LED whose wavelength is 1310 nm as light source, a photodiode, SPR sensors, an SNMP agent, and an SNMP Manager.

#### 3.1.2. System Verification and Results

**Figure 4.**
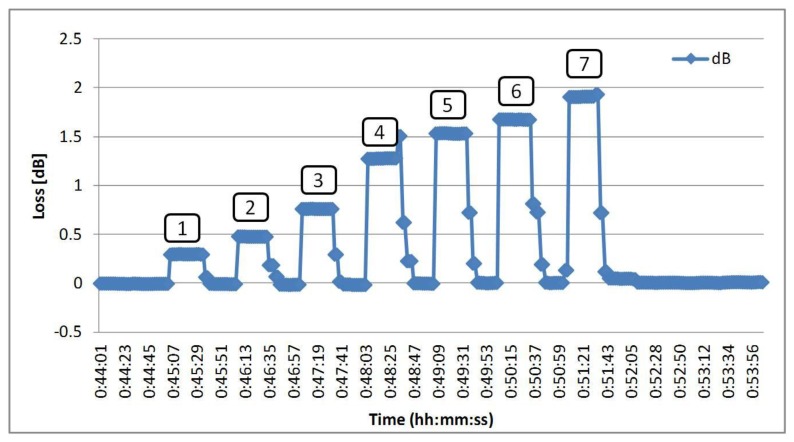
The loss value of sensors measured using SNMP.

The experiment was conducted with the test patterns listed in Table 1. An “ON” signal means that the sensor is immersed in water. The loss value of sensors immersed in water is captured using the SNMP agent, and the value is calculated. The results are shown in Figure 4.

The results showed that each combination pattern could detect a different loss value. Therefore, this value is use to set the threshold value in the SNMP agent and configure the MIB settings. Each combination pattern is assigned to a trap number in the SNMP manager. When a sensor is immersed into water, the trap numbers will be shown according to the response of the sensors. Table 1 shows the results of the experiment. All test patterns were successfully detected.

### 3.2. Identification of Bending Sensors

#### 3.2.1. System Configuration

**Figure 5.**
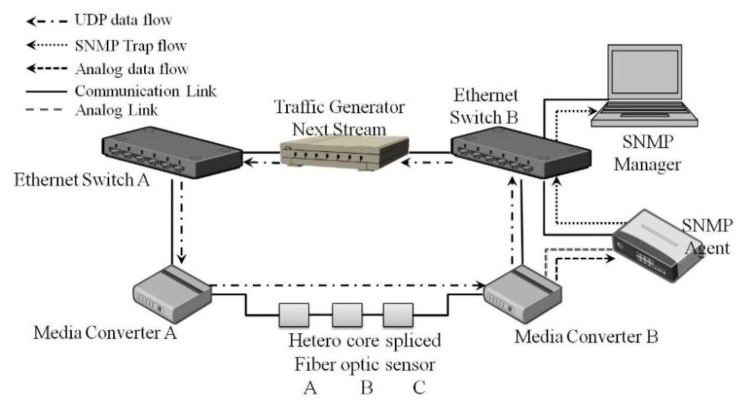
System configuration of sensing system management by SNMP for bending sensors.

The system configuration for remote management by SNMP is shown in Figure 5. The system was configured with a UDP traffic generator (Next Stream NX 6000F, Fujitsu Kyushu, Network Technologies, Japan), Ethernet switch (Dell PowerConnect 5324, Dell, Round Rock, TX, USA), a media converter, an SNMP agent (Asabi), and a monitoring PC. The HC bending sensor, configured as a binary switch module as described in Section 2.1.2, was used in this verification experiment. When the binary switch module push button is pressed, the sensor is in the “ON” state, which decreases the light intensity; that is, the ON/OFF status of the sensor directly affects the light intensity. By adjusting the settings for measurements, the response from each sensor can be detected and distinguished.

In this study, the converted voltage value from the media converter is used as the measurement value. The voltage-measuring instrument in the SNMP agent stores the converted voltage value in the MIB.

Three binary switch sensors are used in the verification experiment. In order to distinguish the input from the sensors, a threshold value is set from each sensor's ON/OFF value by writing a script in the voltage-measuring instrument in the SNMP agent. Each threshold value is assigned a trap number that will be used as the ID of the input from sensors.

The verification experiment starts with generating traffic packets from the traffic generator device and the capturing sensor output values by using a SNMP agent. The sensors were tested in the combinations shown in Table 2. The traffic generator also measured the data communication rate and any frame check sequence (FCS) errors that occurred during the experiment.

**Figure 6.**
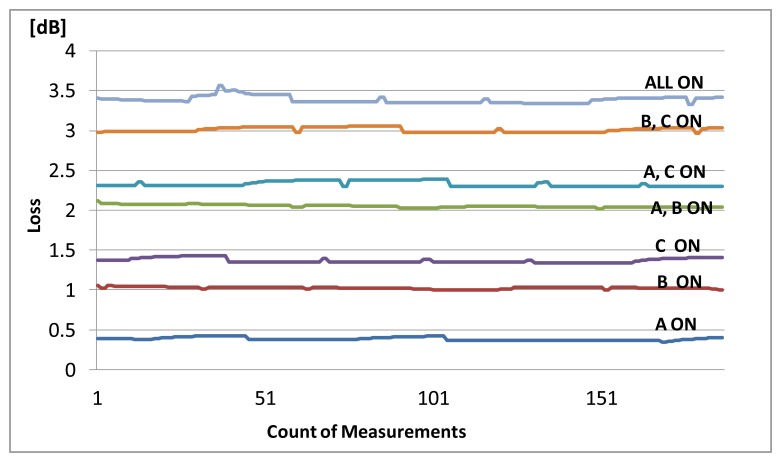
Experiment results for bending sensors.

The experimental results shown in Figure 6 demonstrate that the changes in light intensity from sensors A, B, C, and their combinations can be detected and distinguished. In the SNMP manager, each test pattern is assigned to a trap number; in this way the test results accord to the assigned numbers. In Table 2, the assigned trap numbers for the bending sensors and the detection results are shown. The Next Stream results also verify that there was no communication disturbance and no FCS errors occurred during the experiment.

## 4. Conclusions and Future Works

This study proposed and evaluated the feasibility of remote management for multipoint HC optical fiber sensing systems by SNMP. To distinguish the status of fiber optic sensors and to construct remote data acquisition sensing systems, this studies introduced a method that uses SNMP. According to the experimental verification results, the multipoint HC-SPR sensor sensing system is successfully realized as the responses from each sensor and their combinations can be detected using the proposed method. The bending sensors system verification experiment results demonstrated that data communications and sensing can be successfully achieved in such systems while distinguishing the status of the sensors.

Future work could include developing integrated simultaneous data communication and sensing functions for multiple sensing points on a multimode fiber line. This study need to investigate the “multiplicity boundary” to identify the limit on how many binary switch sensors or HC-SPR sensors can be connected in series under no degradation in communication quality. To expand the usefulness of this type of sensor network, combinations of different types of HC sensors and testing the feasibility of these mixed systems could also be examined.

## Figures and Tables

**Table 1.** Trap number assignment for the SPR sensors and detection results.

**No.**	**Test Pattern Description**	**Assigned Trap Number**	**Result**
1	Sensor 1 ON	Trap No. 32	Detected
2	Sensor 2 ON	Trap No. 33	Detected
3	Sensor 1, 2 ON	Trap No. 34	Detected
4	Sensor 3 ON	Trap No. 35	Detected
5	Sensor 1, 3 ON	Trap No. 36	Detected
6	Sensor 2, 3 ON	Trap No. 37	Detected
7	Sensor 1, 2, 3 ON	Trap No. 38	Detected

**Table 2.** Combination pattern for the bending sensors test and results.

**No.**	**Sensors**			**Assigned Trap Number**	**Results**

	**Sensor A**	**Sensor B**	**Sensor C**		
1	ON	OFF	OFF	Trap No. 21	Detected
2	OFF	ON	OFF	Trap No. 22	Detected
3	ON	ON	OFF	Trap No. 23	Detected
4	OFF	OFF	ON	Trap No. 24	Detected
5	ON	OFF	ON	Trap No. 25	Detected
6	OFF	ON	ON	Trap No. 26	Detected
7	ON	ON	ON	Trap No. 27	Detected
